# Leprosy Continues to Occur in Hilly Areas of North India

**DOI:** 10.1155/2016/7153876

**Published:** 2016-01-24

**Authors:** Deepak Dimri, Arti Gupta, Amit Kumar Singh

**Affiliations:** ^1^Department of Dermatology, Veer Chandra Singh Garhwali Government Medical Science and Research Institute, Uttarakhand 246174, India; ^2^Department of Community Medicine, Veer Chandra Singh Garhwali Government Medical Science and Research Institute, Uttarakhand 246174, India

## Abstract

*Background*. The aim of present study was to describe the profile of leprosy patients attending the outpatient department of dermatology in tertiary care hospital in Srinagar, Uttarakhand, North India.* Methodology*. This descriptive retrospective study. Patient data at the time of diagnosis were retrieved onto a predesigned proforma, which concerned the following variables at the time of registration: age, sex, and residence. Newly registered outpatients leprosy cases between 2009 and 2014 were included in the study.* Results*. It was found that 65 were multibacillary leprosy cases. Males constituted 62.8% of all leprosy cases. The majority (83.7%) belonged to the age group of 18–60 years. Of the total 48.8% of the new leprosy cases were from the Pauri district. The leprosy incidence rate in this population was 2.71 per 1000 patients.* Conclusion*. Leprosy still continues to be a communicable disease of concern. The lower incidence in women and children provokes the need to strengthen contact screening, early case detection, and referral activities in the population to sustain elimination.

## 1. Introduction

Hansen's disease, commonly known as leprosy, is a chronic, granulomatous, infectious disease that primarily affects the skin and the peripheral nerves [1]. Leprosy was eliminated as a public health problem from world in 2000 and from India in December 2005 with prevalence rate less than 1 per 10,000 population [2]. According to World Health Organization, the registered prevalence globally at the beginning of 2012 was 181 941 [3]. Leprosy, although quite rare, continues to occur. In India, a total of 1.27 Lakhs new cases were detected during the year 2013-14, which gives Annual New Case Detection Rate of 9.98 per 100,000 population. The prevalence rate of leprosy in India was 0.68 per 10,000 population with 0.86 Lakhs cases on record as on 1st April 2014 [4]. Also studies have reported discordance between NLEP figures and those reported by dermatologists [5].

The foundation for leprosy elimination was the introduction of antibacterial dapsone monotherapy. The World Health Organization (WHO) introduced multidrug therapy in 1981 due to dapsone resistance. Multidrug therapy since its introduction has not changed except for duration of treatment, which is 1 year in multibacillary cases and continues to be 6 months in paucibacillary cases of leprosy [2]. Adequate and timely treatment of leprosy is critical as it is an important cause of preventable disability [6]. There are gray areas in leprosy elimination as the new cases constantly occur across the country. With the above thought, the aim of this study was to describe the profile of leprosy patients attending the outpatient department in tertiary care hospital in Srinagar, Uttarakhand, North India.

## 2. Materials and Methods

This descriptive retrospective study was conducted at the Medical College Hospital, VCSG Government Medical Sciences and Research Institute, Uttarakhand, which is tertiary care teaching hospital in Northern India. Medical records of all leprosy cases registered by self-reporting between 2009 and March 2014 were retrospectively assessed. All cases registered received WHO recommended fixed duration multidrug therapy. The WHO classification of dividing leprosy into PB (≤5 lesions) and MB (>5 lesions) was used to make the diagnosis [7]. In the present the study the number of MB cases was estimated using the Uttarakhand state data of leprosy [4]. Patient data at the time of diagnosis were retrieved onto a predesigned proforma, which concerned the following variables at the time of registration: age, sex, and residence. Newly registered outpatients leprosy cases between 2009 and 2014 were included in the study. Data were entered in Microsoft excel spreadsheet and analysed with SPSS version 17.0 (Chicago, IL, USA). Descriptive analysis was done. Patients less than 18 years of age were considered as children, and proportions were given wherever necessary.

## 3. Results

A total of 47465 new patients were examined between 2009 and 2014 in outpatient department of dermatology in medical college hospital of VCSG Government Medical Sciences and Research Institute. Of the total 129 new cases of leprosy were detected during the year 2009–14. Of the total, 65 were multibacillary leprosy cases. Males constituted 62.8% of all leprosy cases. Of the total 129 new leprosy cases registered in the institute between 2009 and 2014, 9 were child cases up to 18 years of age. The average child proportion over a period of 5 years was 6.9%. The majority (83.7%) belonged to the age group of 18–60 years. The youngest diseased child was in 7–12 years of age. Nearly half (48.8%) of the new leprosy cases were from the Pauri district (Table 1). The leprosy incidence rate in this population was 2.71 per 1000 person years.

The increase in incidence rate with age reflects the age structure of the population. In addition, the relative increase of incidence rate contributed to by young adults, in particular males, reflects their tendency to seek health care easily compared to women and children in hilly terrains. A total of 9 child cases were recorded in less than 18 years of age, indicating the incidence rate of leprosy among children as 0.99/1,000 patient (Table 2). The incidence rate of leprosy among patient by residence shows the increase in incidence rate with distance travelled (Table 3).

## 4. Discussion

The overall observed incidence rate of leprosy among patient attending outpatient department during the study period was 2.71 per 1000. The prevalence rate of leprosy in Uttarakhand as on March 2014 was 0.22 per 10,000 population. The annual new case detection rate in Uttarakhand as in March 2014 was 3.53 per 10,000 population. The present study reported higher incidence rate of leprosy compared to leprosy data of Uttarakhand. The major reason been the present study is a hospital based study (Table 4) [4].

A male preponderance was seen in our study. This was comparable to other studies. The primary reasons for males preponderance were their greater outdoor activity and increased opportunities for health care [1, 8, 9]. The proportion of new child cases of leprosy in present study was less than 10% of new caseload. Majority of pediatric cases of leprosy in our study belonged to the older age group that is above 12 years. Other studies from Delhi, Andhra Pradesh, Karnataka, and Maharashtra also reported a lesser occurrence in children less than 5 years [8–11]. Similar was the finding of a hospital based study conducted in Nepal [12].

The incidence rate by age, sex, and residence illustrates that each of these factors is an important determinant of leprosy risk. The incidence of leprosy was more prevalent among adults than among younger individuals in present study. The high incidence rates among older adults may be interpreted in various ways. It reflects stable incidence rate in the population for several years prior to this study. Moreover, it could be that individuals were infected late in life or incubation periods were very long. In addition, the incidence rates were higher among males than females over 18 years of age. This was contrary to a population-based study in Malawi where the children and females were found to have higher incidence of leprosy [13]. Alternatively, if incidence rates had been declining in the population for many years, the observed high incidence rates among older adults would be attributable to infections contracted many years previously when transmission of* M. leprae* was more intense than at the time of these surveys. There are no data from the past allowing us to discriminate between these explanations. In addition, the shift in new leprosy case detection towards older ages had occurred over time in Norway [14].

The present study found the increase in incidence rate with distance travelled. Primary reason for this increase of incidence rate can be due to lower number of all patients from distant areas. Other causes are due to low income, lack of knowledge, difficult terrains, and lack of proper transport; sick population of difficult terrain do not visit private health institutions for their medical treatment [15, 16].

The present study was conducted at one tertiary health care setting in North India and hence it cannot be generalised to whole of India. Though the profile was stratified by age and sex, it was not possible to classify on socioeconomic structure and clinical and microbiological features. A relatively long incubation period of leprosy and the chances of misdiagnosing indeterminate skin patches in the initial stages may lead to misclassification of cases.

## 5. Conclusion

Leprosy still continues to be a communicable disease of concern. Our study reports the epidemiology of leprosy in tertiary care hospital. The lower incidence in women and children provokes the need to strengthen contact screening, early case detection, and referral activities in the population to sustain elimination.

## Figures and Tables

**Table 1:** Demographic profile of new cases of leprosy in a tertiary care hospital in Srinagar, Garhwal, North India (2009–2014).

Characteristic	Category	Number of cases (%)
Age group	0–18 years	9 (6.9)
	19–60 years	108 (83.7)
	>60 years	12 (9.4)

Sex	Female	48 (37.2)
	Male	81 (62.8)

District	Pauri	63 (48.8)
	Rudraprayag	16 (12.4)
	Chamoli	6 (4.6)
	Tehri	6 (4.6)
	Others	38 (29.6)

Total		129 (100)

**Table 2:** Observed incidence rates of leprosy per 1000 patient by sex and age in a tertiary care hospital in Srinagar, Garhwal, North India (2009–2014).

Age category (years)	Total new cases		New leprosy cases		Incidence rate per 1000 patient		
	Female	Male	Female	Male	Female	Male	Total
<18	4065	5002	5	4	1.23	0.79	0.99
18–60	17789	17569	41	67	2.30	3.8	3.05
>60	1199	1841	2	10	1.66	5.43	3.94

**Table 3:** Observed incidence rates of leprosy per 1000 patient by district in a tertiary care hospital in Srinagar, Garhwal, North India (2009–2014).

District	Total new case	New leprosy cases	Incidence rate per 1000 patient
Pauri	23527	63	2.67
Rudraprayag	2314	16	6.91
Chamoli	1119	6	5.36
Tehri	2453	6	2.44
Others	18052	38	2.11

Total	47465	129	2.71

**Table 4:** Statistics of leprosy in Uttarakhand as in March 2014 [4] compared with present study.

S. no.	Variable	Uttarakhand (as in March 2014) [4]	Present study (2009–14)
1	Total population (*n*)	10663516	47465^*∗*^
2	Total no. of districts (*n*)	13	5
3	Total cases of leprosy (*n*)	237	129
4	Proportion of multibacillary cases (%)	119	65
5	Annual new case detection rate per 10000 population	3.53	5.4

^*∗*^Patients.

## Abbreviations

NLEP: National Leprosy Elimination Programme
MB: Multibacillary
PB: Paucibacillary.
